# 单中心181例广泛期小细胞肺癌的二线化疗及生存分析

**DOI:** 10.3779/j.issn.1009-3419.2013.11.02

**Published:** 2013-11-20

**Authors:** 满姣 马, 孟昭 王, 燕 徐, 克 胡, 慧慧 刘, 龙芸 李, 巍 钟, 力 张, 静 赵, 华竹 王

**Affiliations:** 1 100730 北京，中国医学科学院北京协和医院呼吸内科 Department of Respiratory Medicine, Peking Union Medical College Hospital, Chinese Academy of Medical Sciences, Beijing 100730, China; 2 100730 北京，中国医学科学院北京协和医院肿瘤放疗科 Department of Oncological Radiotherapy, Peking Union Medical College Hospital, Chinese Academy of Medical Sciences, Beijing 100730, China

**Keywords:** 广泛期小细胞肺癌, 二线化疗, 生存, 影响因素, Extensive-stage small cell lung cancer, Second-line chemotherapy, Survival, Prognostic factors

## Abstract

**背景与目的:**

小细胞肺癌（small cell lung cancer, SCLC）是恶性程度极高的神经内分泌肿瘤，对放化疗敏感，但大多数接受一线化疗的患者在1年-2年内复发。一旦疾病复发，预后不良。目前，广泛期SCLC的一线标准化疗方案为铂类联合依托泊苷方案，二线化疗方案尚无定论。该研究分析二线化疗预后的影响因素；比较不同二线化疗方案的近期疗效、不良反应和远期生存情况的差异。

**方法:**

回顾性分析接受二线化疗的181例广泛期SCLC患者的临床资料。根据不同化疗方案分为6组，采用χ^2^检验比较计数资料和各组组成差异。采用*Kaplan-Meier*法分析总生存时间（overall survival, OS）和无进展生存时间（progression-free survival, PFS）。采用单因素和*Cox*回归多因素分析方法分析生存期的影响因素。

**结果:**

二线化疗的中位OS为7.0个月，单因素和多因素的分析结果表明吸烟情况（*P*=0.004）、ECOG评分（*P* < 0.001）、肝转移（*P*=0.019）和骨转移（*P*=0.028）是影响二线化疗OS的独立影响因素；二线化疗的中位PFS为3.0个月，吸烟（*P*=0.034）、ECOG评分（*P*=0.011）和骨转移（*P*=0.005）是二线化疗PFS的独立影响因素。6组有效率有明显差异（χ^2^=12.413, *P*=0.017），两两比较各组无明显差异；副反应方面，伊立替康组胃肠道反应的发生率明显高于其它组；6组的OS和PFS无统计学差异（χ^2^=1.491, *P*=0.914; χ^2^=6.133, *P*=0.293）。

**结论:**

ECOG评分是影响广泛期SCLC二线化疗预后的最重要因素。广泛期SCLC的二线化疗尚无疗效确切的最优方案。

## Abstract (en)

**Background and objective:**

Small cell lung cancer (SCLC) is the most malignant neuroendocrine tumor and sensitive to chemotherapy and radiotherapy. However, most patients who receive first-line chemotherapy will relapse within one to two years. Once recurrent, it indicates poor prognosis. Currently, the standard first-line chemotherapy regimen of extensive-stage SCLC is platinum combined etoposide regimen while the standard second-line chemotherapy regimen is open to debate. The aim of this study is to analysis the prognostic factors of second-line chemotherapy in extensive-stage SCLC and to compare the differences of objective response rate, side effects and survival among different second-line chemotherapy regimens.

**Methods:**

181 patients who were diagnosed as extensive-stage SCLC and received second-line chemotherapy were collected. χ^2^ test was used to analysis the differences of enumeration data and between different groups. *Kaplan-Meier* method was used to calculate the overall survival (OS) and progression-free survival (PFS). Univariate analysis and *Cox* regression analysis were used to detect the prognostic factors. Objective response rate was evaluated by RECIST criteria and side effects were evaluated by WHO criteria.

**Results:**

The patients who received second-line chemotherapy can be divided into 6 groups, namly group A (CE/EP regimen) 27 cases, group B (regimens containing TPT) 44 cases, group C (regimens containing CPT-11) 33 cases, group D (regimens containing TAX/DXL) 20 cases, group E (regimens containing IFO) 28 cases and group F (other regimens) 29 cases. The median OS in second-line chemotherapy as 7.0 months and was relevant with smoking history (*P*=0.004), ECOG PS (*P* < 0.001), liver metastasis (*P*=0.019) and bone metastasis (*P*=0.028) independently. The median PFS in second-line chemotherapy as 3.0 months and was relevant with smoking history (*P*=0.034), ECOG PS *(P*=0.011) and bone metastasis (*P*=0.005). The response rate among six regimens was significantly different (*P*=0.017); There was not statistical significance between each group. As to side effects, the incidence of gastrointestinal reaction in group C was higher than any other group. The differences of OS and PFS between six regimens in second-line therapy were not statistically different (*P*=0.914, *P*=0.293).

**Conclusion:**

The most significant prognostic factor of extensive-stage small cell lung cancer patients who received second-line chemotherapy was ECOG PS. The most optimal second-line chemotherapy regimen with definite curatice effect was controversial.

小细胞肺癌（small cell lung cancer, SCLC）是恶性程度极高的神经内分泌肿瘤，约占所有肺癌的15%^[1]^。由于具有倍增时间短、易早期转移、侵袭性强等特点而与非小细胞肺癌（non-small cell lung cancer, NSCLC）在分期和治疗方法上不同，预后远不如NSCLC。初诊时约60%-70%的SCLC病人属于广泛期，30%-40%属于局限期。广泛期SCLC中位生存时间约13个月，5年生存率为6.5%^[2, 3]^。SCLC对放化疗敏感，其中化疗是广泛期SCLC治疗的基石，放疗起到辅助治疗的作用，由于SCLC细胞原发或继发耐药，大多数患者在1年-2年内复发^[4]^。一旦疾病复发，广泛期SCLC的中位生存时间大约为4个月。根据患者对化疗敏感性的不同可分为敏感复发、耐药复发和难治性复发。根据复发的时间可以预测二线化疗的疗效和指导选择化疗方案。二线治疗的有效率和缓解期均不如一线化疗，耐药复发者的有效率约10%，敏感复发者的有效率约为25%。但是二线化疗可以延长总生存时间（overall survival, OS）和无进展生存时间（progression-free survival, PFS）、减少肿瘤负荷^[5, 6]^，故出现疾病进展者，若体能状态好，应进行二线化疗。对复发的广泛期SCLC可能有效的药物包括拓扑替康、氨柔比星、伊立替康、吉西他滨、紫杉醇、多西他赛、口服依托泊苷、异环磷酰胺、长春瑞滨等。其中TPT的研究最充分，目前TPT是唯一被美国FDA批准的用于治疗复发SCLC的药物。另一个被批准用于二线治疗的药物是氨柔比星（于日本）。目前，广泛期SCLC的一线标准化疗方案为铂类联合依托泊苷方案，二线化疗方案尚无定论。本研究分析影响二线化疗预后的因素以及比较不同二线化疗方案的近期疗效、不良反应和远期生存情况的差异，旨在为临床治疗广泛期SCLC和判断预后提供一定的参考依据。

## 1 资料与方法

### 1.1 研究对象

收集2001年1月-2011年12月在北京协和医院住院治疗的SCLC患者的临床资料。所有患者需符合以下条件：①年龄大于18周岁；②经细胞学或组织病理学检查确诊为SCLC；③经胸腹部增强CT、全身骨扫描、头颅增强MRI检查，根据美国退伍军人肺癌研究组（Veterans Administration Lung Study Group, VALG）制定的分期方法判定为广泛期SCLC；④疾病进展后接受二线化疗；⑤病历资料完整。

### 1.2 研究方法

收集患者完整的临床资料，包括：患者的年龄、性别、吸烟情况和ECOG评分；病理获取时间、分期、转移部位；化疗方案、最佳疗效、疾病进展时间；末次随访时间及死亡时间。采用电话形式随访，随访时间至患者死亡时间或2013年4月15日为止，存活时间以月为单位。

### 1.3 评定标准

根据实体瘤疗效评价标准（Response Evaluation Criteria in Solid Tumor, RECIST）评估疗效，分为完全缓解（complete response, CR）、部分缓解（partial response, PR）、稳定（stable disease, SD）、进展（progressive disease, PD）。有效率（response rate, RR）=（CR+PR）/全部病例数^*^100%。化疗副反应根据WHO统一标准进行评定。敏感复发是指初始治疗有效且初始治疗结束到疾病进展的时间 > 90天；耐药复发是指初始治疗结束到疾病进展的时间≤90天。OS是指从二线化疗开始至患者死亡或末次随访时间（月）。PFS指从二线化疗开始至疾病进展或患者死亡的时间（月）。

### 1.4 统计学处理

采用SPSS 18.0软件进行数据统计分析。采用χ^2^检验比较计数资料和各组组成差异。采用*Kaplan-Meier*法计算患者的OS和PFS，并绘制生存曲线。采用单因素以及*Cox*回归多因素分析各种因素对生存期的影响。以*P* < 0.05认为差异有统计学差异。

## 2 结果

### 2.1 一般情况

查阅于2001年1月-2011年12月在北京协和医院住院治疗的SCLC患者的临床资料802例，根据实验对象入选标准，符合条件的广泛期SCLC患者共394例，369例接受化疗，181例在疾病进展后接受了二线化疗（49.1%）。全组患者年龄20岁-93岁，中位年龄为60岁。病理组织学上，经典SCLC 178例，混合癌3例。患者一般情况见表 1。

**1:** 广泛期小细胞肺癌二线化疗患者的一般情况 Clinical characteristics of patients in extensive-stage small cell lung cancer patients who received second-line chemotherapy

Characteristics		*n*	Proportion (%)
Sex			
	Male	147	81.2
	Female	34	18.8
Age (yrs)			
	≥60	92	50.8
	＜60	89	49.2
Smoking history			
	Yes	148	81.8
	No	33	18.2
ECOG PS in second-line chemotherapy			
	0-1	146	80.7
	2-4	35	19.3
Brain metastasis			
	Yes	34	18.8
	No	147	81.2
Liver metastasis			
	Yes	34	18.8
	No	147	81.2
Bone metastasis			
	Yes	60	33.1
	No	131	66.9
Adrenal metastasis			
	Yes	31	17.1
	No	150	82.9
Malignant pleural fluid			
	Yes	38	21.0
	No	143	79.0
ECOG PS: Eastern Cooperative Oncology Group performance status.			

### 2.2 治疗情况

181例患者的二线化疗方案可以分为6组，包括：A组（CE/EP方案）27例（14.9%），B组（包含TPT的方案）33例（18.2%），C组（包含CPT-11的方案）44例（24.3%），D组（包含TAX/DXL的方案）20例（11.1%），E组（包含IFO的方案）28例（15.5%），F组（其他方案组）29例（16.0%），其中TAX+IFO方案8例归入了D组。其他方案组方案包括COME/CODE方案7例、口服VP-16方案6例、力比泰方案4例、VM26+BCNU/CBP方案4例、长春瑞滨（NVB）+DDP/CBP方案3例、CAV（CTX+ADM+VCR）方案2例、氨柔比星+DDP方案1例、VP-16+VCR方案1例、索坦1例。一线化疗疗效为PR和CR者122例（67.4%）、SD者41例（22.7%）、PD者12例（6.6%）和疗效不明者6例（3.3%）。耐药复发者（113例）、敏感复发者（43例）、6个月以上复发者（25例）的有效率分别为8.0%、16.3%、20.0%，三者无统计学意义（χ^2^=4.36, *P*=0.101）。6组患者在性别、年龄、吸烟史、ECOG评分、脑转移、肝转移、骨转移、肾上腺转移和恶性胸腔积液的组成上仅性别有明显差异（χ^2^=15.40, *P*=0.009），具有可比性。

### 2.3 二线化疗的OS及其影响因素分析

二线化疗的中位OS为7.0个月，95%CI为5.40月-8.66月。单因素分析的结果（表 2）表明二线化疗的OS与性别、吸烟情况、二线化疗ECOG评分、肝转移、骨转移、复发时间相关；而多因素分析的结果（表 3）表明吸烟情况、ECOG评分、肝转移、骨转移是影响二线化疗OS的独立影响因素。单因素分析和多因素分析结果均提示二线化疗OS与化疗方案的关系无统计学差异。

**2:** 广泛期小细胞肺癌二线化疗总生存期的单因素分析 Univariate analysis of overall survival (OS) in extensive-stage SCLC patients who received second-line chemotherapy

Factors		OS (month)	95%CI (month)	*χ*^2^	*P*
Sex				4.059	0.044
	Male	6.2	4.35-8.05		
	Female	10.7	7.84-13.56		
Age (yrs)				1.040	0.308
	≥60	7.2	4.81-9.65		
	＜60	6.9	5.09-8.71		
Smoking history				6.252	0.012
	Yes	5.9	4.41-7.45		
	No	9.4	4.46-14.41		
ECOG PS				23.233	＜0.001
	0-1	8.2	6.21-10.25		
	2-4	2.7	1.43-4.03		
Brain metastasis				1.772	0.183
	Yes	6.8	5.24-8.42		
	No	7.7	5.89-9.57		
Liver metastasis				6.284	0.012
	Yes	5.7	3.60-7.86		
	No	7.7	5.08-10.38		
Bone metastasis				10.263	0.001
	Yes	4.7	3.89-5.45		
	No	9.1	6.90-11.24		
Adrenal metastasis				0.589	0.443
	Yes	5.9	0.00-12.16		
	No	7.2	5.55-8.85		
Malignant pleural fluid				0.494	0.482
	Yes	9.3	6.23-12.43		
	No	6.8	5.18-8.48		
Second-line chemotherapy regimen				1.491	0.914
	Group A	11.0	0.00-31.43		
	Group B	7.9	6.53-9.33		
	Group C	6.8	5.25-8.41		
	Group D	4.7	4.11-5.23		
	Group E	6.2	0.00-14.53		
	Group F	7.2	5.40-9.75		
Curative effect of first-line chemotherapy				6.342	0.096
	PD	5.9	0.00-11.95		
	SD	6.8	4.96-8.70		
	PR+CR	7.7	5.24-10.22		
	Unknown	1.3	0.00-3.41		
Recurrence status				6.769	0.034
	Resistant	6.8	5.38-8.28		
	Sensitive	7.0	4.06-10.00		
	≥6 months	14.1	5.54-22.66		
Group A：CE/EP regimens; Group B: regimens containing TPT; Group C: regimens containing CCPT-11; Group D: regimens containing TAX/DXL; Group E: regimens containing IFO; Group F: other regimens. PD: progressive disease; SD: stable disease; PR: partial response; CR: complete response.					

**3:** 广泛期小细胞肺癌二线化疗OS的多因素分析 Multivariate analysis of OS in extensive-stage SCLC patients who received second-line chemotherapy

Factors		HR	95%CI	Wald	*P*
Smoking history	Yes	2.03	1.25-3.30	8.169	0.004
ECOG PS	2-4	3.09	1.96-4.88	23.498	﹤0.001
Liver metastasis	Yes	1.62	1.08-2.41	5.478	0.019
Bone metastasis	Yes	1.66	1.06-2.61	4.848	0.028
Compare with patients who did not smoke, whose ECOG performance status was 2-4, who were absence of liver metastasis and bone metastasis, respectively.						

### 2.4 二线化疗PFS及其影响因素分析

二线化疗的中位PFS为3.0个月，95%可信区间为2.53月-3.41月。单因素分析的结果（表 4）表明广泛期SCLC患者二线化疗的PFS与吸烟、ECOG评分、骨转移、一线化疗疗效相关，但多因素分析的结果（表 5）显示吸烟、ECOG评分、骨转移是二线化疗PFS的独立影响因素。

**4:** 广泛期小细胞肺癌二线化疗无进展生存期的单因素分析 Univariate analysis of progression-free survival (PFS) in extensive-stage SCLC patients who received second-line chemotherapy

Factors		PFS (month)	95%CI (month)	*χ*^2^	*P*
Sex				0.634	0.426
	Male	2.8	2.21-3.33		
	Female	3.6	2.64-4.62		
Age (yrs)				0.039	0.844
	≥60	2.8	2.28-3.26		
	< 60	3.1	2.28-3.92		
Smoking history				4.702	0.030
	Yes	2.6	1.99-3.27		
	No	3.9	2.81-4.99		
ECOG PS				7.937	0.005
	0-1	3.1	2.61-3.60		
	2-4	1.5	1.07-1.99		
Brain metastasis				0.126	0.722
	Yes	2.8	1.41-4.13		
	No	3.0	2.40-3.54		
Liver metastasis				0.571	0.450
	Yes	3.0	1.42-4.64		
	No	3.0	2.48-3.47		
Bone metastasis				7.424	0.006
	Yes	1.8	1.10-2.44		
	No	3.1	2.51-3.69		
Adrenal metastasis				0.119	0.730
	Yes	2.6	1.62-3.58		
	No	3.0	2.48-3.46		
Malignant pleural fluid				0.136	0.713
	Yes	2.6	1.77-3.49		
	No	3.0	2.43-3.51		
Second-line chemotherapy regimen				6.133	0.293
	Group A	3.6	1.17-6.09		
	Group B	3.1	2.11-4.16		
	Group C	3.0	1.80-4.13		
	Group D	1.9	1.24-2.60		
	Group E	2.8	0.93-4.61		
	Group F	2.2	0.70-3.76		
Curative effect of first-line chemotherapy				8.348	0.039
	PD	1.9	0.42-3.38		
	SD	2.2	1.49-2.85		
	PR+CR	3.1	2.40-3.86		
	Unknown	0.8	0.73-0.82		
Recurrence status				2.925	0.232
	Resistant	2.8	1.81-3.731		
	Sensitive	2.8	2.11-3.43		
	≥6 months	4.1	2.25-6.01		
Group A：CE/EP regimens; Group B: regimens containing TPT; Group C: regimens containing CCPT-11; Group D: regimens containing TAX/DXL; Group E: regimens containing IFO; Group F: other regimens.					

**5:** 广泛期小细胞肺癌二线化疗无进展生存期的多因素分析 Multivariate analysis of progression-free survival in extensive-stage SCLC patients who received second-line chemotherapy

Factors		HR	95%CI	Wald	*P*
Smoking history	Yes	1.58	1.04-2.42	4.507	0.034
ECOG PS	2-4	1.71	1.13-2.58	6.451	0.011
Bone metastasis	Yes	1.63	1.16-2.29	7.828	0.005
Compare with patients who did not smoke, whose ECOG performance status was 2-4 and who were absence of bone metastasis, respectively.					

### 2.5 6组近期疗效分析

53例（29.3%）因只行1个-2个疗程化疗未进行疗效评估。进行疗效评估者中，PR为11.6%（21/181），SD为21.5%（39/181），PD为37.6%（68/181）。6组的有效率分别为25.9%（7/27），2.3%（1/44），21.2%（7/33），10.0%（2/20），7.1%（2/28），6.9%（2/29），有明显差异（χ^2^=12.41, *P*=0.017）。进一步行两两比较，A组和B组比较差异最为明显（*P*=0.004），而α'=0.05/（C_k_^2^+1）（其中k=6）=0.05/16=0.003125，故各组有效率无明显差异。

### 2.6 6组不良反应分析

113例（占62.4%）患者出现不良反应，常见的不良反应包括骨髓抑制和胃肠道反应（包括恶心、呕吐、腹泻）。骨髓抑制的发生率为49.2%（89/181），其中3度-4度骨髓抑制的发生率为29.3%（53/181），胃肠道反应的发生率为29.3%（53/181）。6个化疗组骨髓抑制的发生率分别为59.3%、54.5%、69.7%、40.0%、32.1%和31.0%，有明显差异（χ^2^=14.91, *P*=0.011），胃肠道反应的发生率分别为18.5%、31.8%、66.7%、10.0%、17.9%和17.2%，有明显差异（χ^2^=31.31, *P*=0.001）。进一步行两两比较，α'=0.05/（C_k_^2^+1）=0.003125。结果显示C组和F组在骨髓抑制的发生率有明显差异（χ^2^=9.24, *P*=0.000, 2），其他各组比较无明显差异；C组与A组、B组、D组、E组和F组在胃肠道反应上均存在显著性差异（*P*=0.000, 2, 0.000, 2, < 0.000, 1, 0.000, 1, 0.000, 1），其他各组比较无显著性差异。

## 3 讨论

本研究中二线化疗的中位OS为7.0个月，中位PFS为3.0个月。据回顾性研究报道，广泛期SCLC二线化疗的中位OS为6个-7个月，中位PFS约为2个-3个月^[7]^，与本研究的结果一致。单因素和多因素分析的结果表明二线化疗的OS和PFS的均与吸烟情况、ECOG评分和骨转移有关。本研究中ECOG评分为0分-1分者的OS和PFS分别为8.2个月和3.1个月，而2分-4分者分别为2.7个月和1.5个月，ECOG评分2分-4分的患者的死亡风险较ECOG评分为0分-1分者提高了2倍，两者相比有显著性差异（*P* < 0.001），故ECOG评分是影响二线化疗生存情况最重要的因素。Korkmaz等^[8]^进行的回顾性分析同样得出ECOG评分是影响SCLC二线化疗重要的影响因素。分析结果还表明吸烟情况是影响二线化疗OS的独立影响因素。据文献^[9]^报道不戒烟确实可以导致化疗药物耐药，疾病复发。目前尚无文献表明骨转移是影响二线化疗生存的重要因素，但有文献报道骨转移是SCLC预后不良的因素之一，可能的原因是骨转移引起的疼痛和骨折使患者的日常生活活动能力（activity of daily living, ADL）下降，患者未能坚持规律化疗所导致的^[10]^。

二线化疗耐药复发者、敏感复发者、6个月以上复发者的有效率分别为8.0%、16.3%、20.0%，三者无统计学意义（χ^2^=4.36, *P*=0.101）。OS的单因素分析结果表明耐药复发和敏感复发患者的OS分别为6.8和7.0个月，两者无统计学意义（*P*=0.15），≥6个月复发患者的OS为14.1个月，与耐药复发相比两者有统计学意义（*P*=0.017）。PFS的单因素分析结果表明耐药复发、敏感复发和≥6个月复发患者的PFS为2.8个月、2.8个月、4.1个月，两两比较三者无统计学意义（*P*=0.38, *P*=0.11, *P*=0.40）。综合上述结果，敏感复发和耐药复发的有效率、OS和PFS无明显差异，≥6个月复发患者的OS和PFS高于前两者，故推测之前研究所报道敏感复发患者的生存时间长是因为纳入了复发时间≥6个月的患者，而复发时间大于3个月小于6个月和小于3个月复发患者的生存情况无异。因此，这种分类方法存在一定的弊端，更好的分类方法有待进一步研究。

本研究对二线化疗中6个化疗方案组进行比较，结果说明6组有效率有明显差异，但进一步两两比较，各组有效率无明显差异；副反应方面，含有伊立替康组的胃肠道反应的发生率明显高于其它组；6组的OS和PFS无统计学差异。TPT作为二线化疗的研究最充分，目前TPT是唯一被美国FDA批准的用于治疗复发SCLC的药物，是广泛期SCLC二线治疗最有效的单药之一。1990s的一项Ⅲ期临床试验比较了拓扑替康单药和CAV方案的疗效，两者的客观应答率（24.3%*vs*18.3%）和中位生存时间（25周*vs*24.7周）相似^[11]^。但本研究TPT的有效率仅2.3%，中位OS为7.9个月，中位PFS为3.6个月，并没有显示出优越性，甚至有效率为6组最低。分析可能存在的原因如下：①患者对拓扑替康的耐受性较差：拓扑替康治疗小细胞肺癌的标准剂量为1.4 mg·m^-2^·d^-1^，但在实际治疗中患者常出现3度-4度血红蛋白和血小板减低，故常需减量治疗，故可能影响TPT的疗效。②国人与欧美人药物遗传背景不同，对药物的敏感性不同；44例TPT组患者有25例仅化疗1个-2个疗程，无法评估疗效。③疗效评估存在偏差。

虽然近30年对SCLC的了解不断加深、有新的化疗药物出现，但广泛期SCLC患者生存情况的改善程度有限，SCLC一线化疗的标准方案仍是EP方案，目前尚无疗效确切的二线化疗最优方案，我们对于疾病的理解并没有转化成可以改善预后的方案和新药。所以，还应继续加强SCLC的基础研究和临床研究，探索治疗SCLC的新策略、新药物，以控制SCLC的发生、发展，改善这种疾病的预后。希望在不久的将来SCLC可以进入预后和生活质量明显改善的恶性肿瘤的名单中。
